# Awareness, Attitudes, and Contraceptive Acceptance Among Women Attending a Tertiary-Care Center in Delhi: A Cross-Sectional Study

**DOI:** 10.7759/cureus.117429

**Published:** 2026-10-04

**Authors:** Suman Lata Mendiratta, Suman Dath S, Ankita Sinha

**Affiliations:** 1 Obstetrics and Gynaecology, North DMC Medical College and Hindu Rao Hospital, Delhi, IND; 2 Obstetrics and Gynaecology, Pravara Institute of Medical Sciences, Maharashtra, IND

**Keywords:** birth spacing, contraception, contraceptive acceptance, contraceptive counseling, family planning, reproductive health

## Abstract

Background: High contraceptive awareness does not always translate into favorable attitudes or actual uptake, particularly when family influence, misconceptions, and concerns about adverse effects shape reproductive decisions.

Objective: This study aimed to assess family planning awareness, attitudes, contraceptive acceptance, and factors associated with acceptance among women attending the gynecology outpatient department of a tertiary-care center in Delhi.

Methods: This descriptive cross-sectional study included 556 women aged 18-48 years who attended the gynecology outpatient department from June 2024 to May 2025 and received family planning counseling. Data were collected using a structured, interviewer-administered data-collection proforma. Categorical variables were summarized as frequencies, percentages, and Wilson 95% confidence intervals. Associations with contraceptive acceptance were assessed using Pearson’s Chi-square test, and effect size was estimated using Cramér’s V.

Results: Overall, 82.0% of participants were aware of family planning, but only 36.5% had a positive attitude. Following counseling, 313 women (56.3%; 95% CI 52.1-60.4) accepted a contraceptive method. Among acceptors, oral contraceptive pills (40.6%), condoms (28.8%), and copper intrauterine contraceptive device (IUCD) (17.3%) were chosen. Doctors were the principal source of information for 37.1% of participants. Husband or family opposition accounted for 80.7% of non-acceptance. A favorable opinion toward birth spacing was reported by 68.0%. Acceptance was significantly associated with age group (χ²=58.90, p < 0.001; Cramér’s V = 0.325) and obstetric category (χ² = 13.50, p = 0.009; Cramér’s V = 0.156), but not with educational status (χ² = 6.29, p = 0.099).

Conclusion: Despite high awareness, a substantial gap between awareness and contraceptive acceptance persisted. Person-centered counseling addressing misconceptions and family-related barriers may improve informed and voluntary contraceptive acceptance.

## Introduction

Family planning is a fundamental component of sexual and reproductive health and an important strategy for improving maternal, neonatal, and child health. Access to safe, acceptable, and affordable contraceptive information and services enables individuals and couples to determine the number and timing of their children, avoid unintended pregnancies, and achieve healthy birth spacing. It also supports women’s autonomy and the health and development of families and communities. Globally, approximately 164 million women of reproductive age have an unmet need for contraception [1]. Common barriers include limited access or method choice, fear or previous experience of adverse effects, misinformation, sociocultural or religious opposition, partner-related constraints, and inadequate quality of counseling and services [1].

India was the first country to launch a National family planning program in 1952 [2]. The program has progressively evolved from a predominantly population-control approach toward a rights-based strategy centered on reproductive choice, informed consent, quality of care, and a broad contraceptive method mix [1,2]. The National Family Health Survey (NFHS-6) reported that the national contraceptive prevalence rate increased from 66.7% in NFHS-5 to 69.1%, while the total fertility rate remained 2.0 [3,4].

In the National Capital Territory of Delhi, NFHS-5 reported use of any contraceptive method among 76.4% of currently married women and use of a modern method among 57.7% [5]. Population-level indicators describe overall program coverage but do not fully explain why individual women accept, postpone, or decline contraception when counseled in clinical settings.

Awareness does not invariably translate into favorable attitudes or actual use. Women may know that contraceptive methods are available yet remain reluctant to adopt them. Studies from different Indian settings have repeatedly demonstrated this knowledge-practice gap and the influence of family, reproductive intentions, perceived adverse effects, and accessibility of services [6-16].

Published evidence remains relatively limited from urban public-sector tertiary hospitals in North India regarding the combined relationship between sociodemographic characteristics, awareness, attitudes, contraceptive acceptance, and reasons for non-acceptance among women attending routine gynecology services. The present study aimed to assess family planning awareness, attitudes, contraceptive acceptance following counseling, and factors associated with acceptance among women attending the gynecology outpatient department.

## Materials and methods

A descriptive cross-sectional study was conducted in the Department of Obstetrics and Gynaecology at North DMC Medical College and Hindu Rao Hospital, Delhi, from June 2024 to May 2025. Consecutive sampling was used: every eligible woman aged 18-48 years who attended the gynecology outpatient department during the study period and provided written informed consent was enrolled. Women younger than 18 or older than 48 years, pregnant women, and women attending for infertility were excluded. Data were collected using the structured, study-specific proforma included in the approved protocol. It recorded demographic and socioeconomic characteristics, obstetric and contraceptive history, awareness and attitude toward family planning, the principal source of information, and views on birth spacing. Awareness was classified as yes or no from the question asking whether the participant had ever heard of family planning. Attitude toward contraceptive use was recorded using the three response categories specified in the study proforma: positive, negative, or not sure. The principal source of information was recorded as one category. Each participant received individual counseling from a doctor regarding available contraceptive methods as part of routine outpatient care. Counseling included discussion of the participant’s contraceptive preferences and willingness to accept a method. The method selected by acceptors and the reasons for non-acceptance were recorded. Contraceptive acceptance was defined as selecting a method at the counseling encounter. Women who declined a method or remained undecided at that encounter were grouped as non-acceptors for analysis. Subsequent initiation and continued use were not assessed. The contraceptive methods discussed in this study are described in the WHO family planning handbook and related medical eligibility and practice guidance [17-19].

Sample size

The minimum sample size was calculated using the formula for estimating a single population proportion:

$n = \frac{Z^{2}pq}{d^{2}}$

where *Z *= 1.96 at a 95% confidence level, *p *is the anticipated prevalence of modern contraceptive use, *q *= 1-p, and *d* is the absolute precision. Based on the NFHS-5 estimate of 57.7% modern contraceptive use among currently married women in Delhi [5], *p* is rounded to 0.58, and *q *is 0.42. Using an absolute precision of 4.3%, the calculated sample size is 506.12 and is rounded up to 507 participants. A total of 556 eligible women were included in the final analysis.

Statistical analysis

Data were entered into Microsoft Excel 2021 (Microsoft Corp., Redmond, WA, USA) and analyzed using IBM SPSS Statistics for Windows, Version 26.0 (Released 2018; IBM Corp., Armonk, NY, USA). Categorical variables were summarized as frequencies and percentages. Wilson 95% confidence intervals were calculated for selected proportions. Associations between contraceptive acceptance and age group, educational status, and obstetric category were assessed using Pearson’s chi-square test. The magnitude of association was estimated using Cramér’s V. All expected cell frequencies exceeded five. A two-sided p-value < 0.05 was considered statistically significant.

Ethical considerations

The study was approved by the Scientific Committee of Hindu Rao Hospital, Delhi, at its meeting held on April 26, 2024 (Ref. No. 3723/MS/HRH; approval letter dated July 9, 2024). Written informed consent was obtained from all participants before enrollment. Participant confidentiality and anonymity were maintained, and data were anonymized before analysis.

## Results

A total of 556 women attending the gynecology outpatient department during the study period were included in the final analysis.

Sociodemographic characteristics

Of the 556 participants, 241 (43.3%) were aged 18-25 years and 219 (39.4%) were aged 26-30 years. Women aged 31-35 years and >35 years constituted 74 (13.3%) and 22 (4.0%) of the study population, respectively. Secondary education had been completed by 336 (60.4%) women, while 109 (19.6%) were graduates or had higher education. Most participants were homemakers (470, 84.5%). Half of the participants belonged to the upper-lower socioeconomic class and 39.2% to the lower-middle class [20].

Obstetric characteristics

The most common obstetric category was two living children (362, 65.1%), followed by one living child (88, 15.8%), three living children (58, 10.4%), and four or more living children (16, 2.9%). Thirty-two participants (5.8%) were in the post-abortal category.

Awareness, attitude, and sources of information

Overall, 456 women (82.0%; 95% CI 78.6-85.0) had heard of family planning, whereas 100 (18.0%) had no prior awareness. A positive attitude was recorded in 203 participants (36.5%), a negative attitude in 292 (52.5%), and uncertainty in 61 (11.0%) (Table 1). Doctors were the principal source of information for 206 participants (37.1%), followed by Accredited Social Health Activist (ASHA) workers or other health personnel (136, 24.5%), and friends or relatives (132, 23.7%) (Table 2).

**Table 1:** Awareness and attitude regarding family planning (N = 556) Data are presented as n, %, and 95% confidence interval (CI).

Variable	Category	n	%	95% CI
Awareness	Yes	456	82	78.6-85.0
	No	100	18	15.0-21.4
Attitude	Positive	203	36.5	32.6-40.6
	Negative	292	52.5	48.4-56.6
	Not sure	61	11	8.6-13.8

**Table 2:** Principal source of information regarding family planning (N = 556) Data are presented as n, %, and 95% confidence interval (CI).

Source	n	%	95% CI
Doctor	206	37.1	33.1-41.1
ASHA worker/other health personnel	136	24.5	21.1-28.2
Friends or relatives	132	23.7	20.4-27.5
Mass media	76	13.7	11.1-16.8
Other	6	1.1	0.5-2.3

Contraceptive acceptance and method choice

Following counseling, 313 women (56.3%; 95% CI 52.1-60.4) accepted a contraceptive method, and 243 (43.7%) did not accept contraception. Among the 313 acceptors, oral contraceptive pills were selected by 127 (40.6%), condoms by 90 (28.8%), copper intrauterine contraceptive device (IUCD) by 54 (17.3%), tubal ligation by 26 (8.3%), and injectable depot medroxyprogesterone acetate (DMPA) by 16 (5.1%) (Table 3).

**Table 3:** Contraceptive acceptance and method selected following counseling Data are presented as n, %, and 95% confidence interval (CI). IUCD: intrauterine contraceptive device, DMPA: depot medroxyprogesterone acetate.

Outcome	n	%	95% CI
Acceptance among all participants (N = 556)			
Accepted	313	56.3	52.1-60.4
Did not accept	243	43.7	39.6-47.9
Method selected among acceptors (n = 313)			
Oral contraceptive pills	127	40.6	35.3-46.1
Condoms	90	28.8	24.0-34.0
Copper IUCD	54	17.3	13.5-21.8
Tubal ligation	26	8.3	5.7-11.9
Injectable DMPA (Antara)	16	5.1	3.2-8.1

Reasons for non-acceptance

Among the 243 women who did not accept contraception, husband or family opposition was the principal reason in 196 (80.7%). Other reasons included fear of side effects (16, 6.6%), desire for future pregnancy (13, 5.3%), other reasons (6, 2.5%), religious beliefs (5, 2.1%), current use of another method (4, 1.6%), and preference for another method (3, 1.2%) (Table 4).

**Table 4:** Principal reasons for non-acceptance among non-acceptors (n = 243) Data are presented as n, %, and 95% confidence interval (CI).

Reason	n	%	95% CI
Husband or family opposition	196	80.7	75.2-85.1
Fear of side effects	16	6.6	4.1-10.4
Desire for future pregnancy	13	5.3	3.2-8.9
Other reasons	6	2.5	1.1-5.3
Religious beliefs	5	2.1	0.9-4.7
Already using another contraceptive method	4	1.6	0.6-4.2
Preference for another contraceptive method	3	1.2	0.4-3.6
Total	243	100.0	-

Opinion regarding birth spacing

A favorable opinion toward birth spacing was reported by 378 participants (68.0%; 95% CI 64.0-71.7), whereas 178 (32.0%) expressed an unfavorable opinion.

Factors associated with contraceptive acceptance

Contraceptive acceptance differed significantly across age groups (χ² = 58.90, df = 3, p < 0.001; Cramér’s V = 0.325), indicating a moderate association. Acceptance was highest among women aged 26-30 years (74.9%), followed by women aged >35 years (72.7%). Educational status was not significantly associated with acceptance (χ² = 6.29, df = 3, p = 0.099; Cramér’s V = 0.106). Obstetric category was significantly associated with acceptance (χ² = 13.50, df = 4, p = 0.009; Cramér’s V = 0.156), although the magnitude of association was small (Table 5).

**Table 5:** Association between participant characteristics and contraceptive acceptance (N = 556) Data are presented as n (%). Associations were assessed using Pearson’s chi-square test. A two-sided p-value < 0.05 is considered statistically significant.

Variable/category	Accepted n (%)	Not accepted n (%)	χ²	df	p-value	Cramér’s V
Age group			58.9	3	<0.001	0.325
18-25 years	104 (43.2)	137 (56.8)				
26-30 years	164 (74.9)	55 (25.1)				
31-35 years	29 (39.2)	45 (60.8)				
>35 years	16 (72.7)	6 (27.3)				
Educational status			6.29	3	0.099	0.106
Illiterate	14 (56.0)	11 (44.0)				
Primary	42 (48.8)	44 (51.2)				
Secondary	203 (60.4)	133 (39.6)				
Graduate and above	54 (49.5)	55 (50.5)				
Obstetric category			13.5	4	0.009	0.156
One living child	47 (53.4)	41 (46.6)				
Two living children	220 (60.8)	142 (39.2)				
Three living children	22 (37.9)	36 (62.1)				
Four or more living children	10 (62.5)	6 (37.5)				
Post-abortal	14 (43.8)	18 (56.2)				

## Discussion

The present study provides contemporary evidence regarding family planning awareness, attitudes, and contraceptive acceptance among women attending a tertiary-care gynecology outpatient department in Delhi. Although 456 (82.0%) women were aware of family planning, only 313 (56.3%) accepted a contraceptive method following counseling, and 292 (52.5%) expressed a negative attitude toward family planning. Lata et al. reported that 550 (55.0%) women agreed to use contraception after counseling, while Singh et al. reported a positive attitude toward contraceptive use among 203 (67.7%) women [7,8].

The majority of participants were young women, with 460 (82.7%) belonging to the 18-30-year age group. Qazi et al. reported 151 (75.6%) participants in the 21-25-year age group and 141 (70.3%) being housewives. Jahan et al. reported 631 (60.1%) participants in the 21-34-year age group [6,10].

Awareness regarding family planning was reported by 456 (82.0%) participants in the present study, whereas Jahan et al. reported awareness in 978 (93.1%) women [10]. The difference between awareness and contraceptive acceptance was one of the most important findings of the present study. Following counseling, 313 (56.3%) women accepted a contraceptive method, while 243 (43.7%) did not accept contraception. Lata et al. reported that 550 (55.0%) women agreed to use contraception after counseling, while Alukal et al. reported prior contraceptive use among 368 (71.6%) women [7,15]. The present study differs from these studies because the primary outcome was acceptance immediately following counseling rather than current or previous contraceptive use.

Acceptance was highest among women aged 26-30 years (164, 74.9%), followed by those aged >35 years (16, 72.7%), whereas it was lower among women aged 18-25 years (104, 43.2%) and 31-35 years (29, 39.2%). The high acceptance among women aged >35 years should be interpreted cautiously because this subgroup included only 22 (4.0%) participants. Basu et al. reported current contraceptive use among 109 (47.8%) women aged ≥25 years compared with 18 (29.0%) among those aged <25 years [13].

Educational status was not significantly associated with contraceptive acceptance in the present study (p = 0.099). Acceptance was numerically highest among women with secondary education (203, 60.4%). In contrast, Singh et al. reported that contraceptive knowledge was significantly associated with educational level, with knowledge present among 130 (100%) women with secondary and above education compared with 14 (45.2%) illiterate women [8].

Acceptance was comparatively high among women with two living children (220, 60.8%), and those with four or more living children (10, 62.5%), while women with three living children had the lowest acceptance (22, 37.9%). The four-or-more subgroup comprised only 16 (2.9%) participants and should therefore be interpreted cautiously. Basu et al. similarly found that current contraceptive use was 102 (47.2%) among women with one or two living children and 24 (57.1%) among those with more than two living children [13].

Among the methods accepted, oral contraceptive pills were most frequently selected (127, 40.6%), followed by condoms (90, 28.8%), and copper IUCD (54, 17.3%). In contrast, Jahan et al. reported condoms as the most commonly used method (430, 65.1%), followed by oral contraceptive pills (210, 31.8%), and IUCDs (60, 9.1%). Maharajan et al. reported Depo-Provera as the most commonly used method (43, 29.5%), followed by condoms (29, 19.9%) [10,21].

Doctors were the principal source of family-planning information in the present study (206, 37.1%), followed by ASHA workers or other health personnel (136, 24.5%). Gupta et al. similarly reported healthcare workers as the major source of long-acting reversible contraception (LARC) information (153, 76.5%). In contrast, Qazi et al. reported media as the principal source of information (90, 45.0%), while Lata et al. reported friends, family, and husband as the source in 400 (40.0%) women [6,7,22].

More than half of the participants had a negative attitude toward family planning despite relatively high awareness. In the present study, 203 (36.5%) women had a positive attitude, whereas Qazi et al. reported a positive attitude among 160 (80.0%) women, and Singh et al. reported a positive attitude among 203 (67.7%) women [6,8]. These differences may reflect variation in study populations, social context, and the instruments used to assess attitude.

Husband or family opposition accounted for 196 (80.7%) among women who did not accept a method at the counseling encounter and was the most prominent barrier identified. Basu et al. reported family opposition to contraception in 68 (23.4%) women and opposition from husbands in 32 (11.0%), with both being significantly associated with contraceptive non-use [13]. The substantially higher proportion of family opposition in the present study highlights the strong influence of interpersonal and household decision-making on contraceptive acceptance and supports the importance of couple-centered counseling when acceptable to the woman [23-25].

Strengths of the study

The study included 556 participants, exceeding the calculated minimum sample size. It evaluated multiple dimensions of family planning, including awareness, attitude, acceptance, method choice, sources of information, reasons for non-acceptance, birth-spacing opinion, and factors associated with acceptance.

Unlike studies limited to contraceptive prevalence, this study examined behavioral and social factors relevant to clinical counseling. Bivariate analyses quantified associations between contraceptive acceptance and age group, educational status, and obstetric category. The tertiary-care setting also provides evidence relevant to similar high-volume urban public hospitals.

Limitations of the study

As a hospital-based cross-sectional study conducted at a single tertiary-care center, the findings may not be generalizable to the wider community, particularly rural populations or women who do not access institutional healthcare. The cross-sectional design precludes assessment of temporal relationships or causality.

Awareness, attitudes, contraceptive decisions, and reasons for non-acceptance were self-reported and may have been affected by recall or social-desirability bias. The study assessed acceptance at the counseling encounter but did not evaluate subsequent initiation, continuation, adherence, discontinuation, method switching, or satisfaction.

Only bivariate associations were assessed; multivariable analysis was not performed. Potential confounding could therefore not be evaluated, and the findings should be described as associated factors rather than independent predictors. Partner education, detailed fertility intentions and household decision-making were not assessed.

Clinical implications

Family planning counseling should be integrated into gynecology, obstetric, post-abortal, and other reproductive-health outpatient visits. Counseling should be individualized according to reproductive intentions, medical eligibility, anticipated side effects, and method-specific concerns. Misconceptions regarding infertility, menstrual disturbances, weight changes, and long-term adverse effects should be addressed using evidence-based information. Partners or family members may be involved when appropriate and with the woman’s consent, while preserving her autonomy and confidentiality. Consistent availability of reversible and permanent options, follow-up, and management of side effects may improve informed choice and continuation. Groups with comparatively lower acceptance may benefit from focused counseling tailored to their reproductive needs and concerns.

## Conclusions

Awareness regarding family planning was high among women attending the gynecology outpatient department; however, a substantial gap persisted between awareness and contraceptive acceptance following counseling. Oral contraceptive pills were the most frequently selected method, while husband or family opposition was the predominant reason for non-acceptance. Healthcare providers, particularly doctors, were the principal source of information. Although more than two-thirds of participants expressed a favorable opinion toward birth spacing, negative attitudes, misconceptions, and family influence continued to hinder acceptance. Age group and obstetric category were significantly associated with acceptance, whereas educational status was not.

Individualized, non-directive counseling, proactive management of concerns about side effects, partner involvement with the woman’s consent, and reliable access to a broad range of methods may improve informed and voluntary contraceptive acceptance.

## Appendices

This appendix summarizes the study-specific data-collection fields and the definitions used in the final analysis. Identifying fields have been omitted.

Participant and clinical details: Age, education, occupation, marital status, socioeconomic status, menstrual history, obstetric history (including number of pregnancies, number of living children, and history of induced abortion), breastfeeding status, medical and surgical history, current medication, contraceptive history, and relevant examination findings.

Family-planning awareness: “Ever heard of family planning?” - Yes / No. Methods of family planning known to the participant were specified where applicable.

Attitude toward contraceptive use: Positive / Negative / Not sure.

Source of information about contraception: For analysis, responses were grouped as doctor, ASHA worker or other health personnel, friends or relatives, mass media, or other.

Contraceptive history: Last contraceptive method used - None / oral contraceptive pill / intrauterine contraceptive device / depot medroxyprogesterone acetate injection / condom / permanent method. Reported side effects, reasons for discontinuing a previous method, and the date of discontinuation were recorded where applicable.

Opinion regarding birth spacing: Responses were presented in the final analysis as favorable or unfavorable.

Counseling decision and analysis definitions: Each participant received individual counseling from a doctor during routine outpatient care. The selected method was recorded for women who chose one at that encounter. For the study analysis, selecting a method at the counseling encounter was classified as acceptance. Declining a method or remaining undecided at that encounter was classified as non-acceptance. The principal reason reported for non-acceptance during counseling was grouped for analysis as husband or family opposition, fear of side effects, desire for future pregnancy, religious beliefs, already using another method, preference for another method, or another reason. Subsequent initiation and continued use were not assessed.
